# Different Contribution of Missense and Loss‐of‐Function Variants to the Genetic Structure of Familial and Sporadic Meniere Disease

**DOI:** 10.1002/mco2.70394

**Published:** 2025-09-21

**Authors:** Alberto M. Parra‐Perez, Alvaro Gallego‐Martinez, Alba Escalera‐Balsera, Paula Robles‐Bolivar, Patricia Perez‐Carpena, Jose A. Lopez‐Escamez

**Affiliations:** ^1^ Otology and Neurotology Group CTS495 Division of Otolaryngology Department of Surgery Instituto De Investigación Biosanitaria ibs.GRANADA Universidad De Granada Granada Spain; ^2^ Sensorineural Pathology Programme Centro De Investigación Biomédica En Red en Enfermedades Raras CIBERER Madrid Spain; ^3^ Deparment of Otolaryngology Instituto De Investigación Biosanitaria ibs.GRANADA Hospital Universitario San Cecilio Granada Spain; ^4^ Meniere's Disease Neuroscience Research Program Faculty of Medicine & Health School of Medical Sciences The Kolling Institute The University of Sydney Sydney New South Wales Australia

**Keywords:** exome sequencing, genetic diagnosis, genomics, Meniere disease, rare variant analysis

## Abstract

Meniere disease (MD) is a chronic inner ear disorder with significant heritability. This study compares the burden of rare high‐ and moderate‐impact coding variants in an MD cohort to determine whether genetic burden in sporadic MD (SMD) overlaps familial MD (FMD), potentially revealing hidden inheritance in SMD. Exome sequencing identified rare variants in unrelated FMD (*N* = 93) and SMD (*N* = 287) patients. Gene Burden Analysis (GBA) was performed, and candidate genes were prioritized using the number of variant carriers, inner‐ear expression, and hearing/balance‐related phenotypic annotations. FMD patients showed higher accumulation of missense and loss‐of‐function variants than SMD, especially in genes linked to auditory and vestibular function. GBA identified 269 enriched genes in SMD, with 31 annotated for inner ear phenotypes, while FMD had 432 with 51 pinpointed. Sporadic and FMD overlapped in 28.1% of enriched genes, with *ADGRV1*, *MEGF8*, and *MYO7A* most commonly shared. Auditory brainstem responses from knockout mouse models supported hearing loss of three novel MD candidate genes (*NIN*, *CCDC88C*, and *ANKRD24*), consistent with patient hearing profiles. In conclusion, SMD and FMD have a divergent genetic architecture. The enrichment of missense variants in stria vascularis and hair cell stereocilia genes supports distinct pathogenic mechanisms and a multiallelic‐recessive inheritance pattern in MD.

## Graphical abstract

This study examines the genetic basis of sporadic (SMD) and familial Meniere disease (FMD) by comparing rare protein‐coding variants using exome sequencing and gene burden analysis. FMD patients had a higher accumulation of missense and LoF variants, especially in genes linked to auditory and vestibular function. Although SMD and FMD share enriched genes, both have genetic architectures suggesting different pathogenic mechanisms and a possible multiallelic‐recessive inheritance pattern.

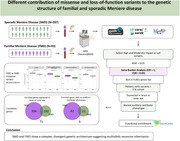

## 1 Introduction

Meniere Disease (MD) is a chronic, progressive inner ear disorder characterized by episodic vertigo associated with a fluctuating sensorineural hearing loss (SNHL), tinnitus, or aural fullness [1]. MD prevalence is population‐specific, being more prevalent in Europeans than in Asians [2]. The MD phenotype is variable, and several clinical subgroups have been defined according to its familial aggregation and associated comorbidities, such as migraine and autoimmune diseases [3]. In addition, an important number of MD patients show a systemic proinflammatory response, which is associated either with high levels of IgE and Type 2 immune response and/or high levels of IL‐1β [4].

It is considered a complex disorder with a strong familial aggregation in Europeans [5]. Familial MD (FMD) was initially reported as an autosomal dominant condition with incomplete penetrance and anticipation in subsequent generations [6], but patients could also show a compound recessive [7] or digenic inheritance pattern [8]. Exome sequencing (WES) has identified several genes related to FMD. Single‐nucleotide variants (SNVs) in *DTNA* (HGNC:3057), *FAM136A* (HGNC:25911), *PRKCB* (HGNC:9395), *DPT* (HGNC:3011), *SEMA3D* (HGNC:10726), *COCH* (HGNC:2180), *GUSB* (HGNC:4696), and *SLC6A7* (HGNC:11054) were identified in different families with an autosomal dominant inheritance pattern with incomplete penetrance. Likewise, genes with a recessive inheritance pattern, such as *HMX2* (HGNC:5018), *LSAMP* (HGNC:6705), and *STRC* (HGNC:16035), have been reported. Nevertheless, no replication of these findings has been found either in other FMD or sporadic MD (SMD) cases, suggesting a considerable genetic heterogeneity [9].

Since these genes were being discovered in individual families, the Gene Burden Analysis (GBA) has allowed the identification of rare variants in the same genes in a broader number of families. This approach has led to the discovery of a burden of rare variation in three SNHL genes in several MD families, which are *OTOG* (HGNC:8516), *MYO7A* (HGNC:7606), and *TECTA* (HGNC:11720) [7, 8, 10], with a recessive, digenic, and dominant inheritance pattern, respectively.

Although significant progress has been made in identifying variants and genes associated with FMD, the genetic underpinnings of SMD remain less studied. Despite the absence of an apparent hereditary pattern in SMD, previous studies reported an enrichment of missense variants in SNHL genes [*GJB2* (HGNC:4284), *USH1G* (HGNC:16356), *SLC26A4* (HGNC:8818), *ESRR* (HGNC:3473), and *CLDN14* (HGNC:2035)] [11] and genes related to axonal guidance signaling [*NTN4* (HGNC:13658)] [12].

In this study, we aimed to compare the distribution of rare variants with a high and moderate impact on the protein in a large cohort of MD patients. In addition, we sought to ascertain whether the genetic burden of SMD diverges or overlaps with FMD to uncover hidden inheritance in sporadic cases.

## 2 Results

### 2.1 Genetic Variants in Sporadic and FMD

The total number of genetic variants obtained was 195,454 and 330,037 in the FMD cohort (*N* = 93) and SMD cohort (*N* = 287 patients) (Table S1), respectively. The distribution of variants with an allelic frequency (AF) < 0.01 was similar between FMD and SMD (Table S2).

**FIGURE 1:**
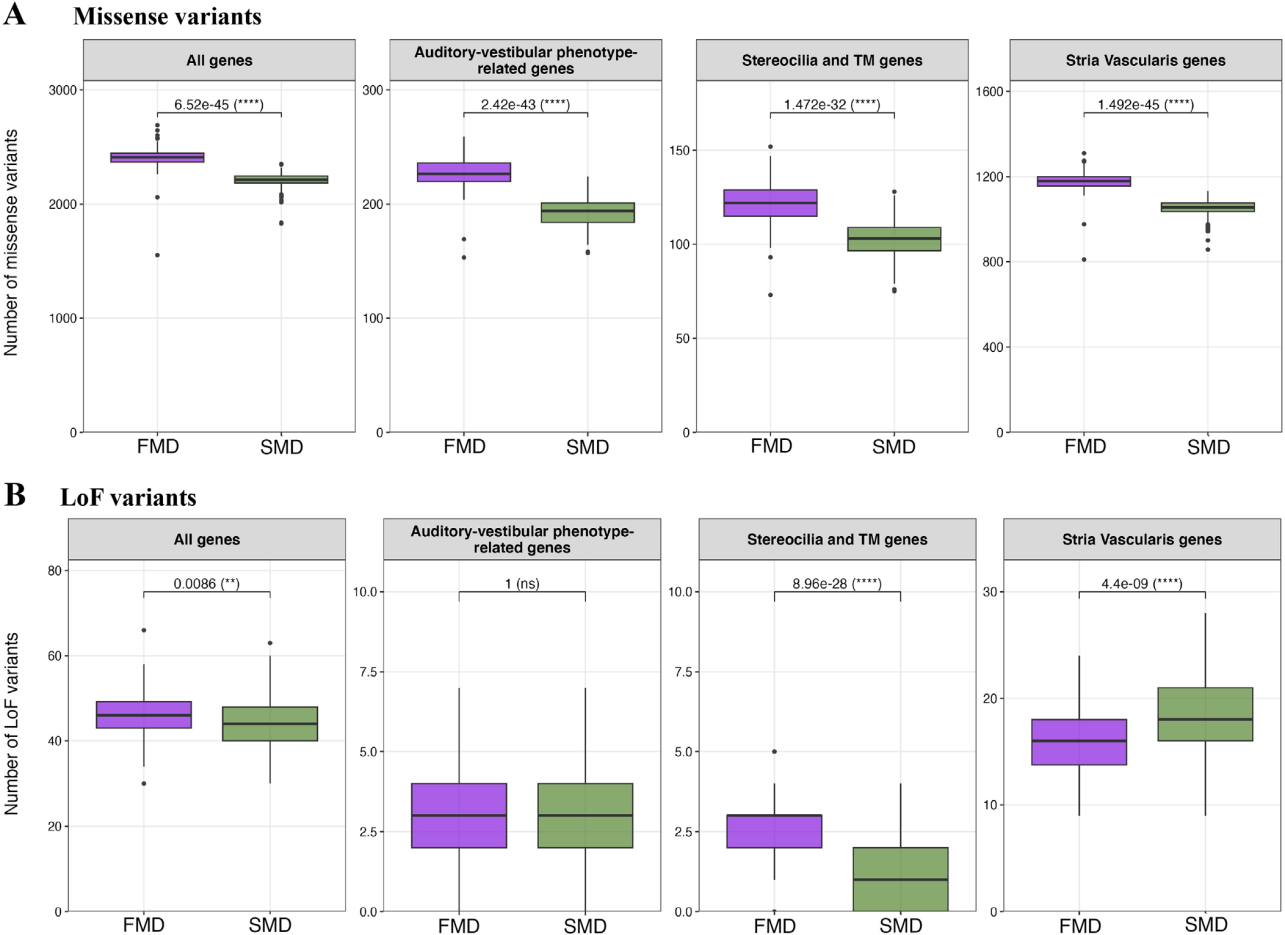
Boxplot comparing the number of missense (A) and loss‐of‐function (LoF) (B) variants in FMD (purple) and SMD (green) patients. The number of total variants in all exome genes, in genes previously associated with an auditory–vestibular phenotype in HPO or MGI, genes expressing proteins present in hair cell stereocilia and tectorial membrane (TM), and genes expressed in stria vascularis are compared from left to right. The Bonferroni‐corrected *p* value obtained with the Mann–Whitney test is shown. Variant counts in a control set of genes, including those associated with cardiovascular disease (CVD), diabetes, and kidney disease, are shown in Figure S1. *p* < 0.05 is considered significant. NS, not significant.

Based on the number of missense variants with an AF < 0.01 per patient, FMD patients showed a significantly higher variant accumulation in all genes (FMD median [IQR] = 2410 [2369.25, 2445.75]; SMD median [IQR] = 2214 [2184.5, 2246]; *p* = 6.52 × 10^−45^), in genes related to an auditory vestibular phenotype (FMD median [IQR] = 226.5 [219.75, 236]; SMD median [IQR] = 194 [184, 201]; *p* = 2.42 × 10^−43^), genes encoding hair cell stereocilia and tectorial membrane (TM) proteins (FMD median [IQR] = 122 [115, 129]; SMD median [IQR] = 103 [96.5, 109]; *p* = 1.47 × 10^−32^), and genes expressed in the stria vascularis (FMD median [IQR] = 1179 [1156. 75, 1200.50]; SMD median [IQR] = 1057 [1037, 1077]; *p* = 1.49 × 10^−45^), compared to SMD patients (Figure 1A).

On the other hand, the number of loss‐of‐function (LoF) variants with an AF < 0.01 was higher in FMD compared to SMD considering all genes (FMD median [IQR] = 46 [43, 49.25]; SMD median [IQR] = 44 [40, 48]; *p* = 8.6 × 10^−3^) and genes encoding stereocilia and TM proteins (FMD median [IQR] = 3 [2, 3]; SMD median [IQR] = 1 [0, 2]; *p* = 8.96 × 10^−28^). Nevertheless, the accumulation of LoF variants in FMD and SMD is similar for genes associated with an auditory and vestibular phenotype and significantly lower for genes expressed in stria vascularis (FMD median [IQR] = 16 [13.75, 18]; SMD median [IQR] = 18 [16, 21]; *p* = 4.4 × 10^−9^) (Figure 1B).

Missense variant accumulation in control gene sets unrelated to MD, including cardiovascular disease (CVD), diabetes, and kidney‐related genes, showed smaller differences between FMD and SMD, with no significant differences for LoF variants (Figure S1).

### 2.2 Enriched Genes in SMD

**FIGURE 2:**
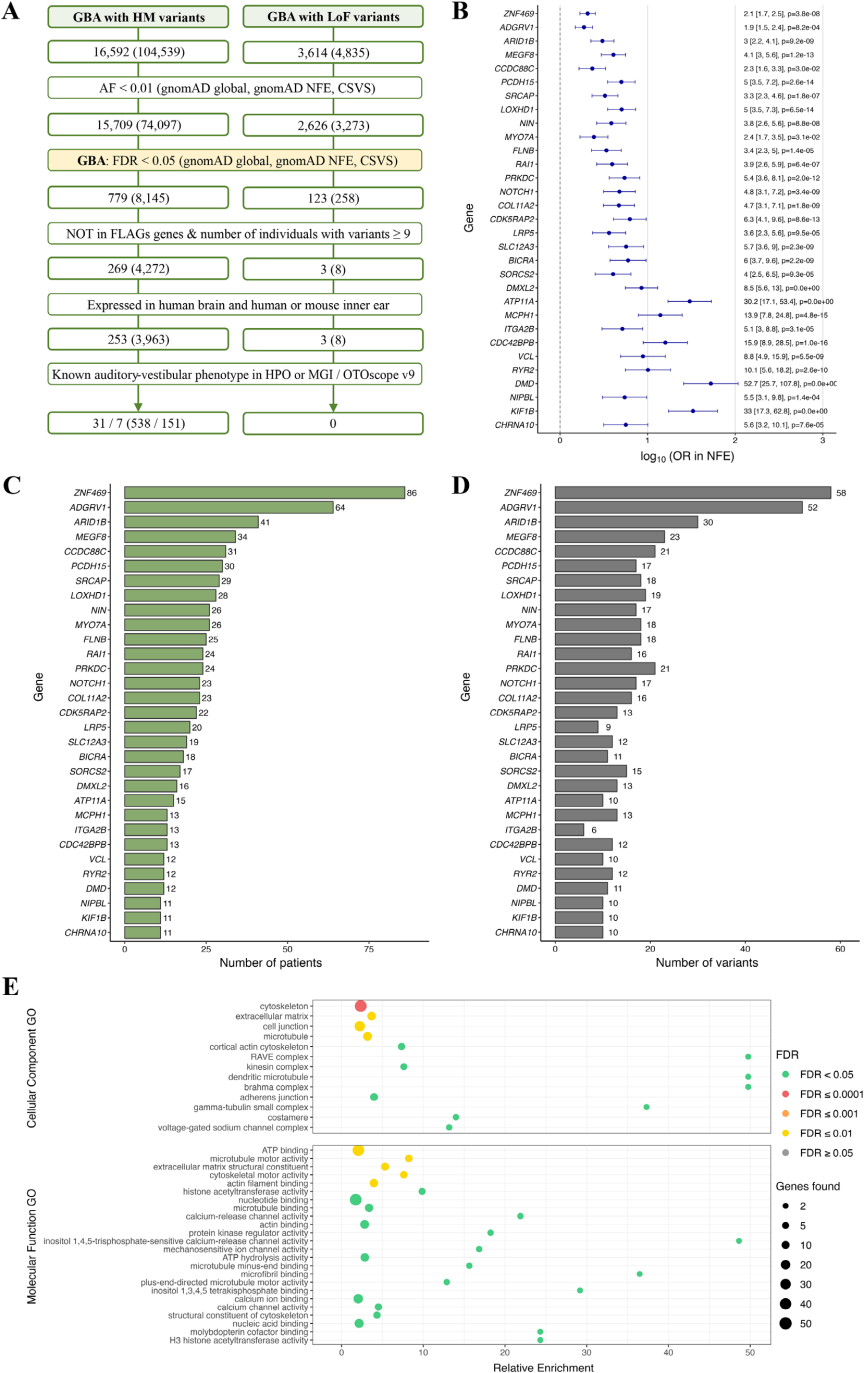
Rare variant analysis and functional enrichment results of candidate genes for the sporadic MD cohort. (A) Flow chart summarizing the prioritization strategy and the result of the GBA for SMD patients, variants filtered by allelic frequency (AF) < 0.01. (B) Odds ratio (OR) and 95% CI, expressed as log10, of the genes associated with an auditory–vestibular phenotype enriched in variants with a high or moderate (HM) impact on the protein and LoF, filtered by AF < 0.01, in the SMD cohort, against gnomAD NFE. Genes were ranked according to the number of individuals with variants. (C) Histogram of the number of SMD patients and (D) number of variants found in genes associated with an auditory–vestibular phenotype enriched in HM and LoF variants. (E) Dot plot representing relative enrichment, FDR‐adjusted *p* value, and number of genes associated with each term using the enriched genes in the SMD cohort expressed in the inner ear. Enrichment analysis was performed using Cellular Components data from the Gene Ontology (GO) database (top) and Molecular Functions data from the GO database (bottom). Red color represents *p* < 0.0001; orange, *p* < 0.001; yellow, *p* < 0.01; and green, *p* < 0.05. CSVS, Collaborative Spanish Variant Server, Spanish population; FDR, *p* value corrected by false discovery ratio; FLAGS, frequently mutated genes; gnomAD NFE, non‐finnish European for gnomAD; gnomAD, Global population for gnomAD; HPO, human phenotype ontology; MGI, mouse genome informatics.

In the SMD cohort, 74,097 variants with an AF < 0.01 and a high or moderate impact on the protein, according to VEP, were found in 15,709 genes. Significant variant enrichment in 779 genes (Table S3), with 8145 variants, was found in the GBA compared to the reference populations (FDR < 0.05). After filtering FLAGS genes and considering genes with variants present in more than 3% of the individuals (*N* > 8 patients), a total of 269 genes (Table S4), with 4272 variants (Table S5), were obtained. Given the large number of potential candidate genes, those with HPO or MGI phenotypic annotations related to the inner ear were prioritized. This resulted in a total of 31 genes (Table S6), with 538 variants (Table S7), among which 7 genes with 151 variants are included in the OTOscope v9 panel used for genetic hearing loss diagnosis (Table S6) (Figure 2A–D). Additionally, 11 of these genes are expressed in hair cell stereocilia (Table S6). Most of the variants identified in the 31 genes were missense (496 variants), with 12 affecting splicing regions. Additionally, 9 frameshift, 4 stop‐gained, 8 in‐frame insertion, and 19 in‐frame deletion variants were found. Notably, nine different patients carried the following variants: NC_000016.10:g.88431891C>T in *ZNF469* (HGNC:26216) and NC_000010.11:g.53961876C>A in *PCDH15* (HGNC:14674).

A total of 270 patients (94% of the SMD cohort) had variants in the 31 enriched genes. Among them, 58 individuals had a unique variant, while the majority had at least two variants in the enriched genes, up to a maximum of eight genes with a variant in one individual. Furthermore, 11 patients had at least 2 variants in *ADGRV1* (HGNC:17416), 5 patients had 2 variants in *MEGF8* (HGNC:3233), 3 in *MYO7A* (HGNC:7606), and 3 patients had 2 variants in *FLNB* (HGNC:3755), *KIF1B* (HGNC:16636), and *PRKDC* (HGNC:9413).

Accounting for possible digenic inheritance, 31 enriched gene pairs with variants were identified in SMD patients. Among these, the *ADGRV1*‐*MYO7A* pair, whose resultant proteins physically interact in hair cell stereocilia, was found in seven patients.

Considering only LoF variants, 3273 LoF variants were found in 2626 genes with a MAF < 0.01. GBA identified 123 genes (Table S8) with 258 variants significantly (FDR < 0.05) enriched in the SMD cohort. After applying the aforementioned filters, three genes (Table S9), with eight variants (Table S10), were defined as candidate genes. The enriched genes, ranked by the number of individuals with variants, were *SYNGAP1* (HGNC:11497), *SKA3* (HGNC:20262), and *BTNL8* (HGNC:26131). None of these genes has phenotypic annotations related to the inner ear in HPO and MGI.

### 2.3 Enriched Genes in FMD

**FIGURE 3:**
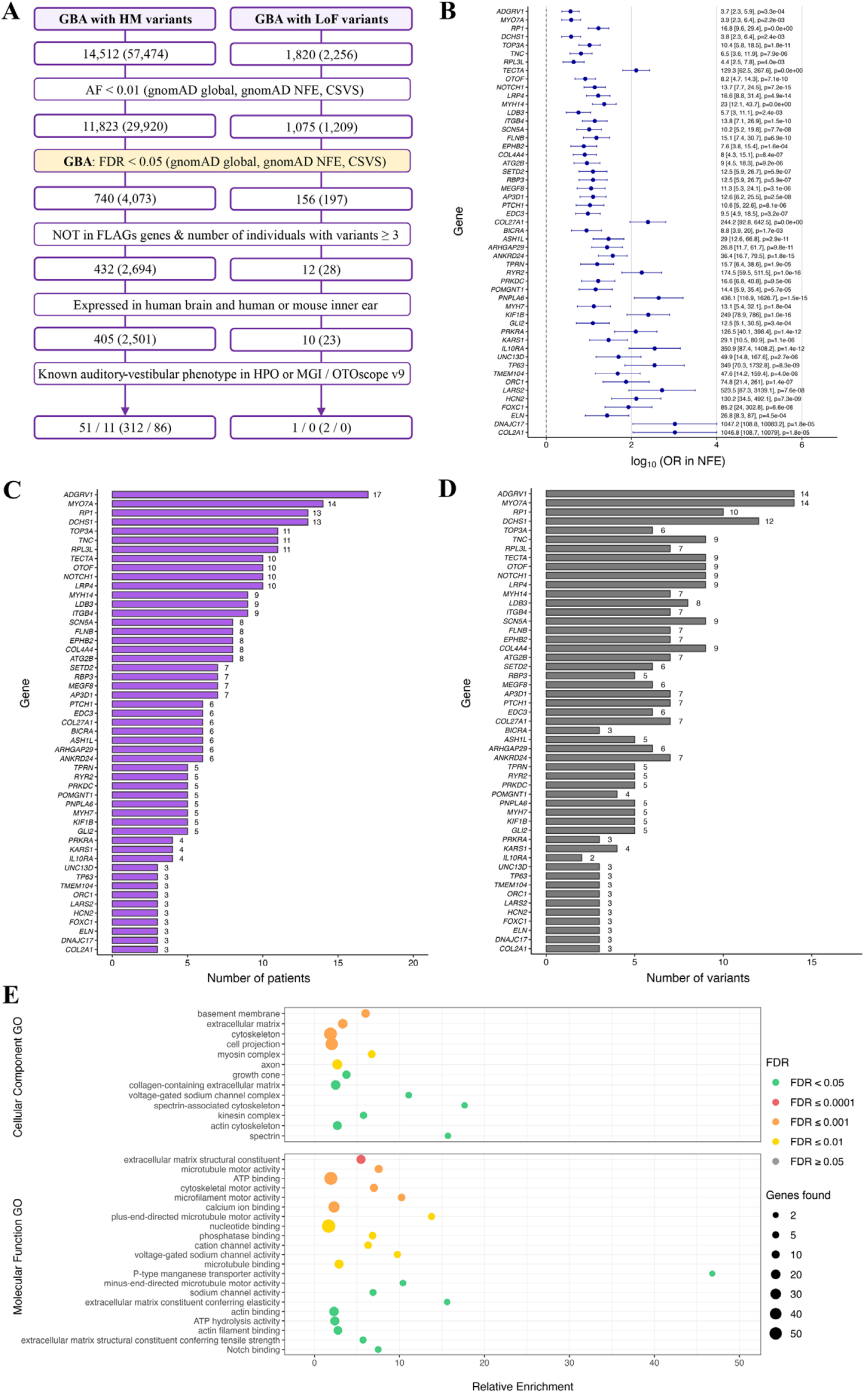
Rare variant analysis and functional enrichment results of candidate genes for the familial MD cohort. (A) Flow chart summarizing the prioritization strategy and the result of the GBA for FMD patients, variants filtered by allelic frequency (AF) < 0.01. (B) Odds ratio (OR) and 95% CI, expressed as log10, of the genes associated with an auditory–vestibular phenotype enriched in variants with a high or moderate (HM) impact on the protein and LoF, filtered by AF < 0.01, in the FMD cohort, against gnomAD NFE. Genes were ranked according to the number of individuals with variants. (C) Histogram of the number of FMD patients and (D) number of variants found in genes associated with an auditory–vestibular phenotype enriched in HM and LoF variants. (E) Dot plot representing relative enrichment, FDR‐adjusted *p* value, and number of genes associated with each term using the enriched genes in the FMD cohort expressed in the inner ear. Enrichment analysis was performed using Cellular Components data from the Gene Ontology (GO) database (top) and Molecular Functions data from the GO database (bottom). Red color represents *p* < 0.0001, orange *p* < 0.001, yellow *p* < 0.01, and green *p* < 0.05. CSVS, Collaborative Spanish Variant Server, Spanish population; FDR, *p* value corrected by false discovery ratio; FLAGS, frequently mutated genes; gnomAD NFE, non‐finnish European for gnomAD; gnomAD, Global population for gnomAD; HPO, human phenotype ontology; MGI, mouse genome informatics.

A total of 28,920 variants, with an AF < 0.01 and high or moderate impact, across 11,823 genes were found in the FMD cohort. GBA showed significant enrichment in 740 genes (Table S11) with 4073 variants. By excluding FLAGs and genes with variants in less than three subjects, 432 genes (Table S12) with 2694 variants were reported (Table S13). The prioritization of genes with inner ear phenotypic annotations yielded 51 genes (Table S14), with 312 variants (Table S15), including 11 genes with 86 variants in the OTOscope v9 panel (Table S14) (Figure 3A–D). Moreover, 13 of these genes were expressed in hair cell stereocilia (Table S14). Most of the variants found in the 51 enriched genes were missense (282 variants), 9 of which affected splice regions. In addition, six frameshift variants, two start lost, one stop lost, two splice acceptors, one splice donor, seven in‐frame deletions, and one in‐frame insertion were found. The variants shared by a higher number of patients, four for each variant, were NC_000005.10:g.90706355A>C in *ADGRV1* (HGNC:17416), NC_000009.12:g.136505868G>A in *NOTCH1* (HGNC:7881), NC_000019.10:g.47698710G>A in *BRICA* (HGNC:4332), NC_00006.10:g.1952959C>T in *RPL3L* (HGNC:10351), NC_000017.11:g.18278232G>A in *TOP3A* (HGNC:11992), and NC_000019.10:g.50266974G>A in *MYH14* (HGNC:23212).

It should be noted that a total of 89 patients (95.7% of the FMD cohort) had variants in the 51 enriched genes. Among these, everyone had 2 variants in the same or different enriched genes, and 71 individuals had 3 or more variants, with one patient having up to 9 variants.

Finally, regarding LoF variants with a MAF < 0.01, a total of 1324 variants in 1075 genes were found in the FMD cohort. After performing GBA, 156 genes (Table S16) with 197 variants were significantly enriched. After filtering steps, including only expressed genes in the inner ear and brain, 10 genes (Table S17) with 23 variants (Table S18) could be considered as potential candidates in the FMD group. The enriched genes, ranked by number of patients with variants, were *SKA3* (HGNC:20262), *BTNL8* (HGNC:26131), *RBM5* (HGNC:9902), *SYNGAP1* (HGNC:11497), *MYO15B* (HGNC:14083), *LPIN3* (HGNC:14451), *CASP10* (HGNC:1500)*, LRP8* (HGNC:6700), *PRKRA* (HGNC:9438), and *SYTL2* (HGNC:15585). The *PRKRA* gene could be highlighted since in the MGI database, it is associated with an increased or absent threshold for auditory brainstem response (ABR) (MP:0011967) and phenotypes related to ear development and temporal bone morphology.

### 2.4 Shared Genes in Familiar and SMD

**FIGURE 4:**
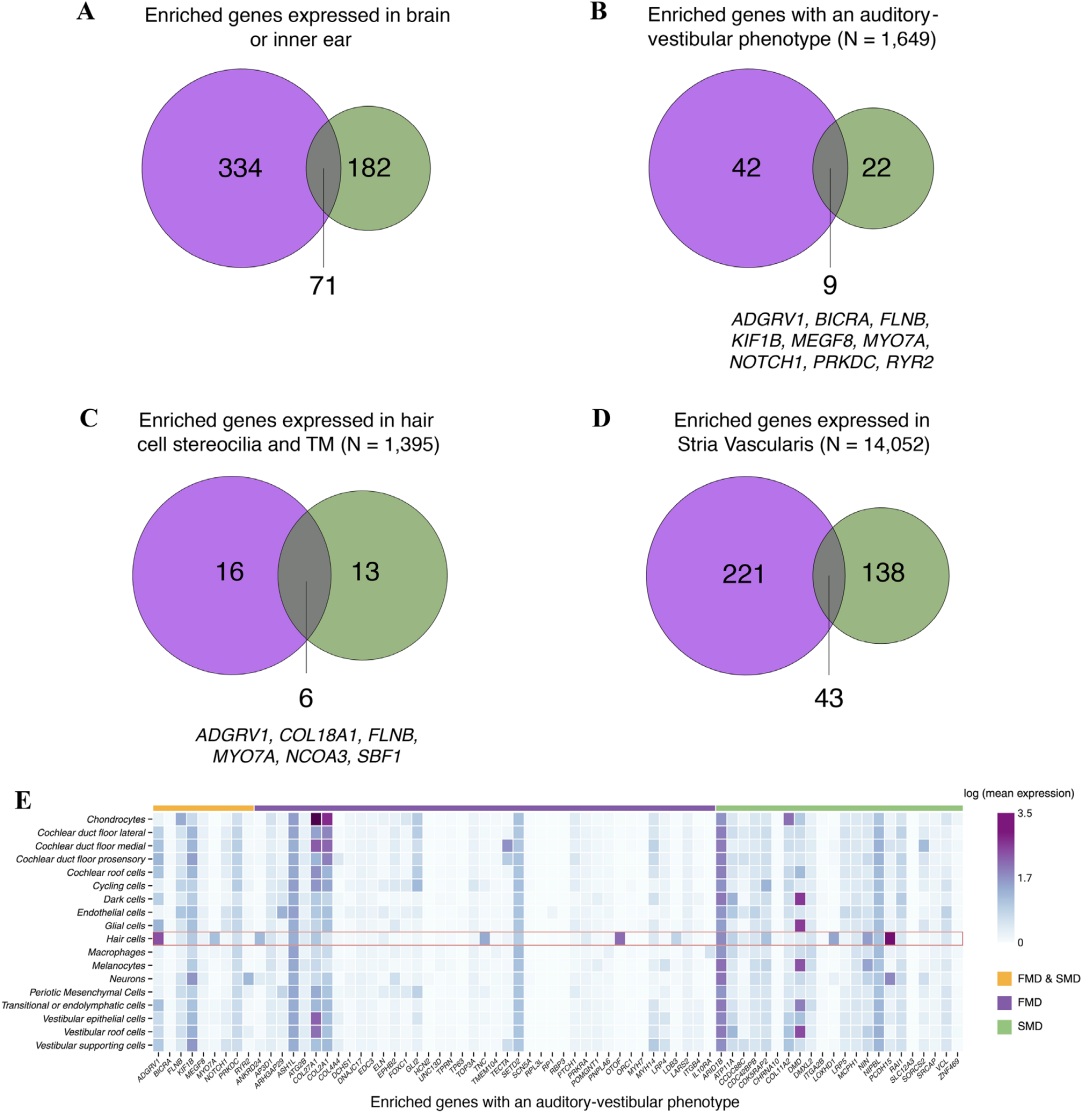
Venn diagrams and gene expression heatmap of enriched genes obtained from the SMD (green) and FMD (purple) GBA. (A) Using all enriched genes expressed in the brain or the inner ear. (B) Using enriched genes expressed in the brain or inner ear and with an associated auditory–vestibular phenotype. (C) Using enriched genes that encode for proteins found in hair cell stereocilia and the tectorial membrane (TM). (D) Using enriched genes expressed in stria vascularis cells. (E) Heatmap of scRNA‐seq expression data from the human inner ear showing the expression patterns of genes included in panel B across inner ear cell types. Created with BioRender.com.

A total of 71 enriched genes were shared in SMD and FMD. In this context, considering the enriched genes expressed in the inner ear and brain, with high and moderate impact variants, 28.1% (71/253) and 17.5% (71/405) of the genes were shared between SMD and FMD (Figure 4A). Considering genes with phenotypic annotations related to the inner ear, 29% (9/31) and 17.6% (9/51) of the enriched genes were shared for SMD and FMD, respectively (Figure 4B). The common enriched genes with phenotypic annotation were *ADGRV1*, *BICRA*, *FLNB* (HGNC:3755), *KIF1B* (HGNC:16636), *MEGF8* (HGNC:3233), *MYO7A*, *NOTCH1*, *PRKDC* (HGNC:9413), and *RYR2* (HGNC:10484).

For the GBA with LoF variants, the enriched genes shared between SMD and FMD were *SYNGAP1*, *SKA3*, and *BTNL8*.

To understand the implications of SMD‐ and FMD‐enriched genes, several enrichment functional analyses were performed. Regarding the functional analysis with the Cellular Component (CC) ontology, 13 significant Gene Ontology (GO) terms (FDR < 0.05) were obtained for both SMD and FMD (Figures 2E and 3E, Tables S19 and S20). The overlapping terms were “*Extracellular matrix*” (GO:0031012), “*Cytoskeleton*” (GO:0005856), “*Voltage‐gated sodium channel complex*” (GO:0001518), and “*Kinesin complex*” in the microtubule (GO:0005871). Using the Molecular Function (MF) ontology of the GO database, 24 and 21 significant terms (*p* < 0.05) were obtained for SMD and FMD, respectively (Figures 2E and 3E, Tables S21 and S22). Of note, the enriched terms include “*Microtubule motor activity*” (GO:0003777), “*Calcium ion binding*” (GO:0005509), and “*Actin filament binding*” (GO:0051015).

### 2.5 Cellular Distribution of MD Genes in the Inner Ear

To explore the cellular distribution of the genes enriched in patients with auditory and vestibular phenotypes (Figure 4B), their expression patterns were analyzed across inner ear cell populations using single‐cell RNAseq data (Figure 4E). Genes from the FMD cohort, such as *ANKRD24*, *OTOF*, and *TNC*, showed high expression in hair cells, similar to genes enriched in the SMD cohort, including *ARID1B*, *LOXHD1*, *NIN*, and *PCDH15*. Shared genes between FMD and SMD cohorts, such as *ADGRV1, KIF1B*, and *MYO7A*, displayed high expression not only in hair cells but also in vestibular epithelial and supporting cells.

Among the enriched genes in the SMD cohort, 19 encode proteins located in hair cell stereocilia and TM (*N* = 1392, 1.36% of the total proteins) (Figure 4C), while 181 genes are expressed in the stria vascularis (*N* = 14,052, 1.29% of the total expressed genes) (Figure 4D). Similarly, in the FMD cohort, 22 genes are expressed in the hair cell bundles and TM (1.72% of the total proteins) (Figure 4C), and 264 genes in the stria vascularis (1.89% of the total expressed genes) (Figure 4D). Variants in stereocilia‐related genes were present in 82.9% (238 out of 287) of SMD patients and 83.9% of FMD patients (78 out of 93). Concerning the enriched genes expressed in the stria vascularis, variants were found in all SMD and FMD patients.

Among them, six enriched genes were shared by both cohorts [*ADGRV1* (HGNC:17416), *COL18A1* (HGNC:2195), *FLNB* (HGNC:3755), *MYO7A* (HGNC:7606), *NCOA3* (HGNC:7670), and *SBF1* (HGNC:10542)] (Figure 4C) and encode proteins present in hair cell stereocilia and TM. In addition, 43 enriched genes, shared between the FMD and SMD, were expressed in stria vascularis cells (Table S23 and Figure 4D).

### 2.6 Functional Validation of Candidate Genes Using ABRs From Knockout Mouse Models

A total of 96 knockout (KO) models corresponding to genes enriched in SMD and 146 KO models for FMD were retrieved from IMPC, of which 17 and 27 genes, respectively, had prior auditory phenotype annotations.

**FIGURE 5:**
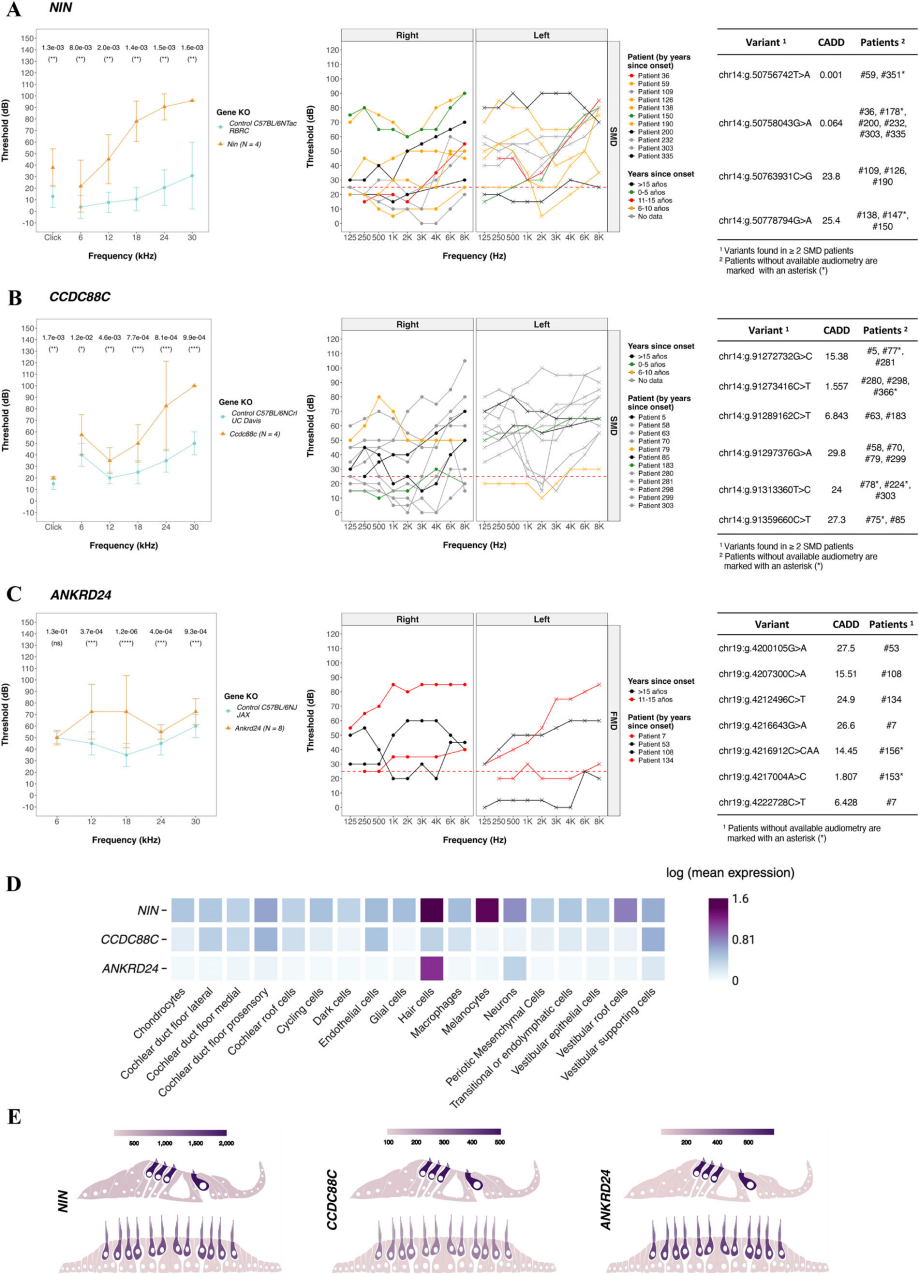
Audiological and expression data for *NIN* (A), *CCDC88C* (B), and *ANKRD24* (C) genes. (A–C) Left panels: Auditory Brainstem Responses (ABRs) in knockout (KO, orange) and control (blue) mice. Middle panels: Audiograms of patients carrying variants in each gene, colored according to disease duration. Right panels: Table of rare variants identified in each gene in the FMD and SMD cohorts. (D) Heatmap of single‐cell RNA‐seq expression data from the human inner ear, showing expression patterns of *NIN*, *CCDC88C*, and *ANKRD24* across inner ear cell types. Created with BioRender.com. (E) Bulk RNA‐seq expression levels of the same genes in inner ear tissues, based on data from Elkon et al. [35].

As expected, KO models for established SNHL genes (*ADGRV1*, *MYO7A*, and *TPRN*) exhibited significant SNHL compared to controls. Beyond these, three novel candidate genes showed SNHL in KO mice: *NIN*, *CCDC88C*, and *ANKRD24*. Notably, *NIN* and *CCDC88C* were enriched in the SMD cohort, with rare variants identified in 26 and 31 patients, respectively, while *ANKRD24* was enriched in the FMD cohort, with variants present in 6 patients. The NIN KO mice showed hearing impairment across all tested frequencies, with mild‐to‐moderate hearing loss at 6 and 12 kHz and more severe at higher frequencies (Figure 5A). Correspondingly, patients with *NIN* variants exhibited moderate‐to‐severe hearing loss, with no correlation to variant CADD scores or disease duration. Similarly, *CCDC88C* KO mice showed hearing loss mainly at high frequencies (Figure 5B), paralleling a patient phenotype characterized by predominantly unilateral left‐ear impairment with several bilateral cases. Finally, *ANKRD24* KO mice showed elevated auditory thresholds at mid‐to‐high frequencies (12, 18, 24, and 30 kHz; Figure 5C), consistent with patient data showing unilateral and bilateral hearing loss.

Single‐cell RNA sequencing revealed that *NIN* is broadly expressed across cochlear and vestibular cell types, with particularly high expression in hair cells, melanocytes, and neurons. *CCDC88C* expression was enriched in hair cells, cochlear duct floor prosensory cells, and vestibular supporting cells, whereas *ANKRD24* was predominantly expressed in hair cells and neurons (Figure 5D,E). These expression patterns were consistent with bulk RNA sequencing data.

## 3 Discussion

Rare variant analysis can facilitate the path to genetic diagnosis in MD. This study compares the distribution of rare variants in SMD and FMD and supports an overload of rare missense and LoF variants that may contribute to MD development. The identification of different and overlapping genes between SMD and FMD confirms a complex genetic architecture and underlines the relevance of understanding both forms to develop more effective molecular diagnostic strategies.

In the SMD cohort, a significant variant enrichment in 779 genes compared to the global, NFE, and Spanish reference populations was identified. After filtering, we found 31 genes with rare variants in more than 9 patients, with phenotypic annotations related to the inner ear. Most of the SMD patients (94%) had rare variants in these 31 genes, suggesting a higher predisposition to suffer MD. Notably, 19 of these genes are expressed in the hair cell stereocilia, highlighting their potential role in the pathophysiology of SMD. In addition, 181 enriched genes are found expressed in stria vascularis cells.

Among the genes enriched in SMD, *ADGRV1*, *PCDH15*, and *MYO7A* stand out for their location at the stereocilia in the tip links and ankle links. Variants in these genes could contribute to a disorganization and lack of coordination in the stereocilia that would lead to abnormal gating of the MET channel, resulting in prolonged hair cell depolarization, vertigo, and hearing impairment [13]. Besides, *MYO7A* and *PCDH15* have been previously associated with FMD [8]. Other noteworthy genes include *LOXHD1*, a gene described for DFNB77, which is located along the plasma membrane of hair cell stereocilia and is required for the mechanotransduction process [14]; *CHRNA10*, which codes for an ionotropic receptor that modulates auditory stimuli and was found associated with hearing loss in the UK Biobank cohort [15, 16]; and *COL11A2*, associated with DFNA53 and DFNB13 [17]. Besides, 16 genes (*ADGRV1, ARID1B, BICRA, CDK5RAP2, COL11A2, DMD, DMXL2, FLNB, LRP5, MEGF8, MYO7A, NIPBL, PCDH15, PRKDC, VCL, ZNF469*) have been previously associated with SNHL in HPO, and 10 are located at cell–cell junctions according to CC GO, supporting the formation of endolymphatic hydrops observed in some MD patients [18, 19].

Similarly, in the FMD cohort, a significant enrichment of variants with a moderate impact was observed in 740 genes. After filtering and prioritizing, 51 enriched genes with inner ear phenotypic annotations were identified. Most of the FMD patients (95.7%) had variants in these 51 genes, also suggesting a high relevance of these genes in the MD development. Among all enriched genes, 22 genes are expressed in hair cell stereocilia and TM, supporting their involvement in FMD under the hypothesis that fragile stereocilia could be destabilized and may provoke vertigo attacks and hearing loss [9]. Besides, 264 genes are found expressed in stria vascularis cells.

Previously, FMD‐associated genes *MYO7A* and *TECTA* were included among the enriched genes [8, 10]. In addition, *ADGRV1, COL2A1, COL4A4, KARS1, LARS2, MYH14, OTOF, TNC*, and *TPRN* have been described for several forms of dominant and recessive hearing loss according to OTOscope v9, and 19 additional genes have been associated with SNHL in HPO.

All FMD and SMD patients had variants with a moderate impact on the enriched genes, which could contribute to the different MD expressivity. This would suggest that SMD patients present a recessive type of inheritance. Furthermore, the presence of multiple variants in those genes in several patients suggests a polygenic inheritance that may contribute to disease variability in both FMD and SMD.

Subsequently, if the genetic architecture of SMD and FMD is compared, an overlap in 71 enriched genes could be observed. Common genes, such as *ADGRV1, BICRA, FLNB, KIF1B, MYO7A, NOTCH1*, and *PRKDC*, could indicate common genetic pathways involved in both forms of the disease and a possible hidden inheritance in SMD. These mechanisms could be related to both an aberrant hair cell stereocilia structure [9] and alterations in the stria vascularis in terms of cell–cell junction [19], for example. However, our results support additional pathogenic mechanisms in SMD and FMD, according to the number of variants and enriched genes found.

The concept of polygenic inheritance in SMD has also been supported in a large cohort of unilateral MD patients (*N* = 527), who reported 481 genes were involved in hearing, balance, and cochlear function, as well as cell–cell adhesion and extracellular matrix organization [19]. Compared with this study, 48 SMD (6 with an auditory–vestibular phenotype) and 56 FMD genes (11 with an auditory–vestibular phenotype) of the total number of enriched genes were replicated. Combining the genes enriched in SMD and FMD, a total of 86 genes were replicated (Table S24). In the functional analysis, pathways related to proteins present in the extracellular matrix and cell–cell junctions were also identified.

The phenotypes observed in KO mice and the expression profiles across cochlear and vestibular cell types reinforce the potential role of several genes in the pathophysiology of MD. Based on functional validation using KO mouse models, three novel candidate genes (*NIN*, *CCDC88C*, and *ANKRD24*) were identified as potentially contributing to both auditory and vestibular phenotypes. These genes converge on essential cellular processes for inner ear sensory epithelium integrity, including cytoskeletal organization and epithelial polarity. *NIN*, encoding ninein, is essential for anchoring microtubule minus‐ends at non‐centrosomal apical sites in polarized epithelial cells, such as pillar cells of the organ of Corti and vestibular supporting cells. By localizing to adherens junctions, it stabilizes apico‐basal microtubule arrays and epithelial polarity. Its loss may disrupt sensory epithelium cohesion and integrity, potentially impairing both hearing and balance [20]. *CCDC88C*, which encodes Daple, regulates cytoskeletal organization and cell polarity through Wnt and GPCR signaling [21]. Daple‐deficient mice show disorganized stereocilia, abnormal apical microtubules, and impaired cilium positioning, leading to hearing loss and disrupted bundle orientation in both cochlear and vestibular hair cells [22, 23]. These findings also support the role of *CCDC88C* in the auditory and vestibular phenotype observed in MD. ANKRD24, a stereocilia rootlet‐associated protein, contributes to stereocilia anchoring by organizing and stabilizing TRIOBP at their insertion point into the cuticular plate, ensuring structural integrity and resilience of hair cells [24]. A frameshift variant in *ANKRD24* caused non‐syndromic hearing loss in humans, which is compatible with the auditory phenotype observed in the MD patients [25].

Our study has several limitations. First, we have used stringent filters to shorten the MD candidate gene list, excluding genes in the FLAGS gene list (*N* = 100) [26]. These genes have a large coding sequence, exhibit lower evolutionary pressure, and have been more frequently associated with rare pathological phenotypes, reporting rare variants possibly with a functional impact [26]. Two genes that could be relevant in the pathogenesis of MD and that show an enrichment of rare variants in both SMD and FMD cohorts were found in the FLAGS list. These genes are *MYO15A* and *USH2A*. In the case of *MYO15A*, 42 variants have been found in 61 patients with SMD and 21 variants in 19 patients with FMD. This gene is associated with DFNB3 and codes for myosin XVa, a protein necessary for the organization of actin in auditory and vestibular hair cells. It plays a role in their maturation and in the formation of stereocilia through the contribution of Whirlin proteins to the upper part of these structures [27]. Regarding *USH2A*, 45 variants have been identified in 64 patients with SMD, and 20 variants in 16 patients with FMD. This gene is implicated in Usher syndrome 2A and codes for usherin, a protein required for the formation of ankle links. These links are formed together with other proteins, including Myosin VIIa, Adhesion G‐protein coupled receptor V1, PDZ domain‐containing protein 7, Vezatin, Whirlin, and Vlgr1. These proteins help connect the growing stereocilia in developing hair cells [28].

Second, this study focuses on exonic variants, which probably does not cover all the potential molecular mechanisms underlying the onset of MD, and hence, the use of whole genomes could help to carry out a deeper investigation of the MD genetic architecture and its sporadic and familial forms.

Third, GBA identifies statistical associations rather than causality. To reduce incidental findings, we focused on genes with known auditory and vestibular function; however, additional functional studies are needed to confirm their pathogenic role in MD.

Finally, it would be interesting to analyze variants in genes involved in the immune response, as they could be contributing to the MD development in those patients whose etiology seems to be autoimmune or autoinflammatory.

## 4 Conclusion

The genetic structure of sporadic and FMD according to the distribution of rare variants is different, with some overlapping genes, such as *ADGRV1* and *MYO7A*. However, SMD and FMD have an overload of missense variants in stria vascularis and hair cell stereocilia genes that suggest a multiallelic, recessive inheritance pattern. The contribution of LoF variants to either SMD or FMD is lower compared to missense variants, but it seems to be more relevant in stria vascularis genes in SMD.

## 5 Materials and Methods

### 5.1 Patient Recruitment

The recruitment of MD patients was based on the diagnostic criteria established by the International Classification Committee for Vestibular Disorders of the Barany Society [1]. A total of 454 patients with MD were recruited in Spain. Among them, 167 were considered as FMD with one or more first‐degree relatives affected by MD from 93 different unrelated families, and 287 as SMD.

The protocol of this study was approved by the local ethics committee (MS/2014/02, Institutional Review Board for Clinical Research, Universidad de Granada, Spain), and all the subjects signed a written informed consent to donate biological samples. The research was performed following the principles of the Declaration of Helsinki, which was revised in 2013.

### 5.2 Exome Sequencing

Sample collection, DNA extraction, and WES were performed following a previously published protocol [8].

### 5.3 Bioinformatic Analysis

The Sarek Nextflow pipeline, included in NF‐Core [29], was utilized to perform the exome reference alignment, mapping to the GRCh38/hg38 human reference genome, base quality score recalibration (BSQR), variant calling, and quality filtering. Genetic variants were called using the HaplotypeCaller tool from GATK [30]. The VCF files were normalized using the *norm* function from BCFtools and filtered according to the criteria employed by the gnomAD database [31]: allele balance (AB) ≥ 0.2 and AB ≤ 0.8 (for heterozygous genotypes only), genotype quality (GQ) ≥ 20, and depth (DP) ≥ 10 (5 for haploid genotypes on sex chromosomes). Afterwards, using the BCFtools *merge* function, an MD variant dataset was created. Subsequently, variant quality filtering was conducted using Variant Quality Score Recalibration (VQSR), which calculated the VQSLOD score. Variants were filtered according to the VQSLOD value, using the first tranche (90), which corresponds to a 90% truth sensitivity threshold. This cutoff was selected to balance sensitivity and specificity, prioritizing the detection of rare variants while reducing false positives.

Variants were annotated using Ensembl Variant Effect Predictor (VEP) [32]. Variants with high or moderate impact on the protein for FMD and SMD, according to VEP classification, were kept separately for further analyses. Two independent databases were used to obtain the variant's AF in reference populations. AF for non‐Finnish European (NFE) population (*N* = 32,299) and for the global population (*N* = 71,702) were retrieved from the gnomAD database v.3.0 [31]; and AF for the Spanish population (*N* = 2071) from the Collaborative Spanish Variant Server (CSVS) [33].

To highlight genes associated with SMD and FMD, a GBA was performed using the SMD and FMD cohort as previously described [34]. Only one individual from each family was selected, whenever possible, according to the lowest age of onset and/or from the last generation. Variants with an AF < 0.01 in the CSVS, gnomAD NFE, and gnomAD global databases were retained.

For each reference population, the GBA was performed independently. For each variant, we retrieve the allele count (AC) and allele number (AN) in both cases and controls. The number of wild‐type (WT) alleles was computed as WT = AN − AC. To obtain a gene‐level estimate, AC and WT were summed across all variants within each gene. The following statistics were calculated:

$$\mathrm{OR}\;=\frac{\mathrm{AC}\;\mathrm{cases}\;\times \;\mathrm{WT}\;\mathrm{control}}{\mathrm{WT}\;\mathrm{cases}\;\times \;\mathrm{AC}\;\mathrm{control}}$$

$$\mathrm{SE}\;=\sqrt{\frac{1}{\mathrm{AC}\;\mathrm{cases}\;}+\frac{1}{\mathrm{AC}\;\mathrm{control}}+\frac{1}{\mathrm{WT}\;\mathrm{cases}}+\frac{1}{\mathrm{WT}\;\mathrm{control}}}$$

$$Z=\frac{\ln\left(\mathrm{OR}\right)}{\mathrm{SE}}$$

The two‐tailed *p* value (*p*) was calculated using the complementary cumulative distribution function (CCDF), with the formula:

$$p=\;\mathrm{CCDF}\left(\left|Z\right|\right)\;\times \;2$$

The *p* was corrected by false discovery rate (FDR), using the total number of genes:

FDR=p×nGenes

Finally, the 95% CI was calculated as follows:

$$\mathrm{CI}\;=e^{\ln\left(\mathrm{OR}\right)\pm \;1.96\times \;\mathrm{SE}}$$

Variants not present in the reference population were considered novel. In these cases, the AC was imputed as 0, and the AN was estimated as the average AN of all variants in the same gene.

Statistically significant genes (adjusted *p* < 0.05) and an OR ≥ 1 in the three comparisons with each reference population were considered enriched in MD.

### 5.4 Gene Prioritization

Genes with a high mutation rate (FLAGS) [26] were removed based on the top 100 genes listed. In addition, genes not expressed in the inner ear or brain were excluded. In order to obtain the genes expressed in the inner ear or in the brain, RNAseq data from several resources was used: (1) RNAseq data from inner ear (hair cells and non‐hail cells) of P0 and adult mice from Expression Analysis Resource (gEAR) database [35, 36, 37], (2) RNAseq data from P30, M9 and M26 mice stria vascularis [38], (3) microarray RNA from P0 mouse Spiral Ganglion Neuron (SGN) and Vestibular Ganglion Neuron (VGN) from Shared Harvard Inner‐Ear Laboratory Database (SHIELD) [39], (4) RNAseq data from three adult human cochleae, an ampulla, a saccule and an utricle from patients without hearing loss [40], and (5) RNAseq data from human brain tissues from Genotype‐Tissue Expression (GTEx) project V8 [41]. In addition, P3–P5 and P21–P25 mouse utricle proteomics data have been used to select those proteins present in hair cell stereocilia [42].

Genes with variants observed in at least 3% of individuals, even if each individual carried a different variant, were selected and ranked for further analysis. In addition, candidate genes in SMD and FMD were prioritized for their phenotypic association with hearing and balance through annotation with the Human Phenotype Ontology (HPO) [43] and Mouse Genomics Informatics (MGI) [44] databases. Genes annotated with any of the terms described in Table S25 were prioritized.

### 5.5 Gene Expression Analysis in the Inner Ear

To study the cellular expression of candidate genes in the auditory and vestibular organs, single‐cell RNA sequencing (scRNA‐seq) data from a published atlas of human adult inner ear tissues were analyzed [45]. Data processing was performed using the Seurat package (v5.2.0) in R. Gene expression profiles were visualized using heatmaps to highlight relative abundance and cellular specificity.

### 5.6 Functional Analysis

A functional enrichment analysis was carried out to discover the predominant localization and MFs related to the candidate genes. The enrichment analyses were performed via a hypergeometric test utilizing the GeneCodis4 tool [46] and the CC and MF databases from GO [47].

### 5.7 ABR Data From IMPC

To validate GBA candidate genes, ABR data were extracted from KO mouse models and WT controls generated by different phenotyping centers within the International Mouse Phenotyping Consortium (IMPC) program (www.mousephenotype.org) [48]. ABR thresholds in KO mice were compared to center‐matched controls across standard auditory frequencies. Genes whose KO resulted in elevated ABR thresholds were prioritized as functionally relevant candidates in the auditory phenotype of MD.

### 5.8 Statistical Analysis

The number of variants identified in each patient was compared between the FMD and SMD cohorts. The normality of the data distribution was assessed using the Shapiro–Wilk test. As the number of variants per patient did not follow a normal distribution, the nonparametric Mann–Whitney test was used. To account for multiple comparisons, the resulting *p* values were adjusted using the Bonferroni correction method. For ABR data, auditory thresholds at each frequency were compared between KO and control mice using also the Mann–Whitney *U* test. A *p* < 0.05 after correction was considered statistically significant.

## Author Contributions

A.M.P‐P. and J.A.L‐E. conceptualized the study. A.M.P‐P., A.G‐M., A.E‐B., P.R‐B., and P.P‐C. carried out the investigation. A.M.P‐P. performed the formal analysis. A.M.P‐P. and A.E‐B. developed the software. J.A.L‐E. supervised the study. A.M.P‐P. and J.A.L‐E. drafted the manuscript. A.M.P‐P., A.G‐M., A.E‐B., P.R‐B., P.P‐C., and J.A.L‐E. revised the manuscript. All authors approved the final manuscript.

## Ethics Statement

This study has been approved by a local ethics committee (MS/2014/02, Institutional Review Board for Clinical Research, Universidad de Granada, Spain). Written informed consent for publication was obtained from all patients. Data have been deidentified for publication. Studies were performed in accordance with the Declaration of Helsinki.

## Conflicts of Interest

The authors declare no conflicts of interest.

## Supporting information




**Table S1**: Demographics of the SMD and FMD cohort regarding sex, MD age of onset, and MD laterality.
**Table S2**: Number of variants annotated by VEP, with an AF < 0.01, according to variant consequence and impact on protein in the SMD and FMD cohort.
**Table S3**: Statistical summary of the genes enriched in variants with a predicted high or moderate impact on the protein filtered by AF < 0.01, for the SMD cohort.
**Table S4**: Statistical summary of the genes enriched in variants with a predicted high or moderate impact on the protein filtered by AF < 0.01, FLAGS genes and number of individuals > 8, for the SMD cohort.
**Table S5**: Variants reported in SMD cohort in enriched genes derived from the GBA of variants with a high or moderate impact, AF < 0.01, filtered by FLAGS genes and number of individuals > 8
**Table S6**: Statistical summary of the genes enriched in variants with a predicted high or moderate impact on the protein filtered by AF < 0.01, FLAGS genes, number of individuals > 8 and genes related with an auditory–vestibular phenotype, for the SMD cohort.
**Table S7**: Variants reported in SMD cohort in enriched genes related with an audiory–vestibular phenotype, derived from the GBA of variants with a high or moderate impact and AF < 0.01.
Table S8: Statistical summary of the genes enriched in LoF variants filtered by AF < 0.01, for the SMD cohort.
**Table S9**: Statistical summary of the genes enriched in LoF variants filtered by AF < 0.01, FLAGS genes and number of individuals > 8, for the SMD cohort.
**Table S10**: Variants reported in SMD cohort in enriched genes derived from the GBA of LoF variants, AF < 0.01 and filtered by FLAGS snad number of individuals > 8.
**Table S11**: Statistical summary of the genes enriched in variants with a predicted high or moderate impact on the protein filtered by AF < 0.01, for the FMD cohort.
**Table S12**: Statistical summary of the genes enriched in variants with a predicted high or moderate impact on the protein filtered by AF < 0.01, FLAGS genes and number of individuals > 2, for the FMD cohort.
**Table S13**: Variants reported in FMD cohort in enriched genes derived from the GBA of variants with a high or moderate impact, AF < 0.01, filtered by FLAGS genes and number of individuals > 2
**Table S14**: Statistical summary of the genes enriched in variants with a predicted high or moderate impact on the protein filtered by AF < 0.01, FLAGS genes, number of individuals > 2 and genes related with an auditory–vestibular phenotype, for the FMD cohort.
**Table S15**: Variants reported in FMD cohort in GBA enriched genes related with an audiory–vestibular phenotype, derived from the GBA of variants with a high or moderate impact and AF < 0.01.
**Table S16**: Statistical summary of the genes enriched in LoF variants filtered by AF < 0.01, for the FMD cohort.
**Table S17**: Statistical summary of the genes enriched in LoF variants filtered by AF < 0.01, FLAGS genes and number of individuals > 2, for the FMD cohort. Gene symbol in bold indicates that the gene has an inner ear‐related phenotype annotation in HPO or MGI.
**Table S18**: Variants reported in FMD cohort in enriched genes derived from the GBA of LOF variants, AF < 0.01 and filtered by FLAGS and number of individuals > 2.
**Table S19**: Enrichment analysis performed using Cellular Components data from the Gene Ontology (GO) database with the 269 candidates genes for the SMD cohort.
**Table S20**: Enrichment analysis performed using Cellular Components data from the Gene Ontology (GO) database with the 432 candidates genes for the FMD cohort.
**Table S21**: Enrichment analysis performed using Molecular Function data from the Gene Ontology (GO) database with the 269 candidates genes for the SMD cohort.
**Table S22**: Enrichment analysis performed using Molecular Function data from the Gene Ontology (GO) database with the 432 candidates genes for the FMD cohort.
**Table S23**: List of enriched genes expressed in the stria vascularis cells shared between SMD and FMD.
**Table S24**: List of SMD, FMD and all MD candidates genes replicated in the cohort of Fisch, K.M., et al., 2024.
**Table S25**: HPO and MGI terms to filter the candidate gene list.
**Figure S1**: Boxplots comparing the number of missense (A) and loss of function (LoF) (B) variants per individual in FMD (purple) and SMD (green) patients for gene sets unrelated to MD. From left to right, the analysis includes: (1) all exome genes, (2) genes associated with CVD (HP:0001626; *N* = 2834), (3) genes associated with diabetes mellitus (HP:0000819; *N* = 379), and (4) genes expressed in kidney cortex (from GTEx; *N* = 9110). Bonferroni‐corrected *p* values from the Mann–Whitney test are shown. A *p* value < 0.05 was considered significant. NS, not significant.

## Data Availability

The annotated VCF files are available at the European Genome‐Phenome Archive (EGA) under controlled access: EGAD50000001682 (familial Meniere disease cohort) and EGAD50000001683 (sporadic Meniere disease cohort). Both datasets are linked to the study EGAS50000001178: Exome Sequencing of Familial and Sporadic Meniere Disease Patients. Any additional data is available from the corresponding author, J.A.L‐E., upon reasonable request.
